# Beyond Scars: Development and Validation of a Four-Dimensional Body Image Score for Women with Breast and Gynecological Cancers - A Tunisian Multicenter Study

**DOI:** 10.12688/f1000research.169080.1

**Published:** 2025-09-03

**Authors:** Narjes karmous, Abdennour Karmous, Refka Bouslimi, Wided Mohamed

**Affiliations:** 1 Gynaecology and Obstetrics department B, Charles Nicolle Hospital, Tunis, Tunisia; 2 University Tunis el Manar, Faculty of Medicine of Tunis, Tunis, Tunisia; 3 Psychiatric department, Razi Hospital, Tunis, Tunisia; 4 Higher school ofHealth Sciences and Techniques of Tunis, Tunis, Tunisia

**Keywords:** Body image, Sexual well-being, Breast cancer, Gynecological cancer, Psychometric validation, Questionnaire development, Quality of life, Women’s health

## Abstract

**Background:**

Body image disturbances in breast and gynecological cancer survivors involve complex interrelationships between physical changes, sexuality, and self-esteem. Existing tools inadequately capture these multidimensional, cancer-specific concerns. This study developed and validated a Four-Dimensional Body Image Score for women with breast and gynecological Cancers (4D-BISC).

**Methods:**

A cross-sectional study was conducted (March–April 2025) across six Tunisian healthcare facilities. Women presenting with gynecological or breast cancers were included. Participants completed the 16-item questionnaire (5-point Likert scale) derived from validated scales (the Female Sexual Function Index (FSFI) and the Body Esteem Scale for Adolescents and Adults (BESAA)) and cancer-specific items. Psychometric properties were assessed via exploratory/confirmatory factor analyses (EFA/CFA), Cronbach’s alpha (α), and correlations with established scales (FSFI, BESAA).

**Results:**

In total, 101 participants were included. CFA confirmed a robust 4-factor structure: Appearance Concerns (α=0.704), Social Exposure (α=0.737), Sexual Desirability (α=0.918) and Self-Esteem (α=0.786). The global score demonstrated excellent reliability. Strong correlations emerged between body image factors and sexual function (e.g., Sexual Desirability and sexual desire: p=0.706 and p<0.001 respectively). Breast cancer patients reported significantly worse body image globally (p=0.003), particularly in social exposure (median=26.7) and sexual desirability (median=30). Marital status and socioeconomic status significantly influenced sexuality-related concerns and self-esteem (p<0.05).

**Conclusion:**

The 4D-BISC is a psychometrically valid tool capturing cancer-specific body image dimensions. It highlights critical vulnerabilities among breast and gynecological cancers cancer patients- and provides a foundation for targeted psychosocial interventions.

## 1. Introduction

Body image and quality of life are deeply intertwined in survivors of breast and gynecological cancers, with the diagnosis and treatment processes often triggering a complex array of physical and emotional disruptions.

Altered appearance due to disfiguring scars, lymphedema, or alopecia, ^1^ distress related to bodily exposure in both clinical and intimate contexts, ^2^ sexual impairment involving dysfunction or grief over fertility loss, ^3^ and a profound erosion of self-esteem ^4^ all contribute to a persistent and multifaceted suffering. These elements do not exist in isolation but interact dynamically, forming a constellation of distress that often endures long after treatment ends. ^5, 6^

Despite this complexity, existing assessment tools fall short in capturing the full scope of these experiences. Generic instruments, such as the Body Exposure during Sexual Activities Questionnaire (BESAQ), lack sensitivity to cancer-specific contexts- failing to capture post-mastectomy bodily exposure anxiety ^7^ or treatment- induced vaginal changes. ^3, 8, 9^ Similarly, oncology-focused quality of life measures like the European Organisation for Research and Treatment of Cancer Quality of Life Questionnaire (EORTC QLQ) tend to compartmentalize issues, missing the critical interconnections between body image, sexuality, and self-worth. ^7^ As a result, clinicians are often left without effective tools to assess these interrelated domains of distress in a holistic manner. ^10^

The impact of these inadequacies extends beyond individual well-being. Up to 73% of survivors report strain in intimate relationships stemming from body-related distress. ^9, 11^ Fertility loss not only exacerbates sexual dysfunction but also fuels deeper identity crises. ^3, 12^ Additionally, chemotherapy has been strongly associated with marked declines in self-esteem, ^1, 13^ highlighting the urgent need for integrated, survivor-centered approaches to psychosocial care.

Despite the growing body of literature, much remains to be done in terms of integrating body image and sexual well-being into routine oncological care. Most existing scales tend to focus either on physical functioning or emotional health in isolation, without fully capturing the complex interplay between body image, self-esteem, and sexual function— especially in women facing the profound physical changes associated with cancer treatment. ^4, 14^

This article was therefore motivated by the need to develop a more comprehensive and integrative tool that bridges this gap. By combining elements from the Female Sexual Function Index (FSFI) ^15^ and the Body Esteem Scale for Adolescents and Adults (BESAA) ^16^— and by incorporating additional dimensions specifically relevant to women undergoing cancer treatment— we aim to provide a novel composite score capable of capturing the full scope of physical, emotional, and relational challenges faced by these patients. Our goal is to offer a resource not only for researchers, but also for clinicians seeking to deliver more personalized and holistic care to women affected by breast and gynecological cancers.

Given the limitations of existing tools and the multifaceted impact of cancer on women’s physical and psychological well-being, the objective of this study was to design and propose a new composite score that integrates key elements of sexual function and body image perception. This score draws on validated scales, namely the FSFI and the BESAA, while also incorporating additional items specifically tailored to the experiences of women with breast and gynecological cancers—dimensions often neglected in traditional scales.

By doing so, we aim to capture more accurately the global burden of disease on patients’ quality of life and provide a practical tool that could support both clinical assessment and future research. This article presents the methodology behind the construction of the Four-Dimensional Body Image Score for women with breast and gynecological Cancers (4D-BISC), as well as its preliminary application in a diverse population of breast and gynecological cancer patients.

## 2. Methods

### 2.1 Study design and setting

This was a cross-sectional study conducted over a two-month period from March 1, 2025, to April 30, 2025. The study took place in 6 Tunisian healthcare facilities of different levels:

Level 2:

• El Yasminet Regional Hospital, Ben Arous
• Jendouba Regional Hospital, Jendouba

Level 3:

• Charles Nicolle Hospital, Tunis
• La Rabta Maternity and Neonatology Center, Tunis
• Salah Azaiez Institute, Tunis

The study was conducted among women with breast and gynecological cancers using a questionnaire.

A self-administered paper questionnaire was used to collect data in a private room at the outpatient clinic. Participants completed questionnaires independently, with research staff available outside the room to clarify item wording if requested. Staff did not interpret items or suggest responses.

### 2.2 Study population

Patients meeting the following criteria were included:

**Inclusion criteria**

- Women presenting with gynecological or breast cancers, regardless of the type of treatment received.

**Exclusion criteria**

- Age < 18 years.
- Women diagnosed with other types of cancer (excluding gynecological or breast cancers).
- Women who developed severe complications or experienced a recurrence during the study, preventing their participation.
- Incomplete questionnaires.
- Refusal or withdrawal of consent at any point during the study.

### 2.3 Development of the composite score

Further methodological information on the EFA and CFA procedures described in this article are available at this link: https://doi.org/10.7910/DVN/ZGECTK. ^17^

**2.3.1 Methodology – Scoring procedure for the 4D-BISC**

The development of the 4D-BISC involved several stages: a literature review, question design, creation of a scoring system (question scoring), and pre-testing.

The composite score was developed based on a comprehensive review of validated instruments in the field of body image and female sexual health, including the BESAA, the Body Image Scale (BSI), and the FSFI. The aim was to assess the impact of medical diagnosis or treatment on women’s perception of their body image.

The final version included 16 items; each rated on a 5-point Likert scale ranging from 1 (“strongly disagree”) to 5 (“strongly agree”). Higher scores indicate a greater negative impact on body image.

**2.3.2 Confirmatory Factor Analysis (CFA)**

A confirmatory factor analysis revealed a four-factor structure, reflecting the following dimensions:

• **F1: Body concern/physical appearance** (Q1 to Q5)
• **F2: Public exposure/perception by others** (Q6 to Q8)
• **F3: Sexuality and desirability** (Q9 to Q12)
• **F4: Self-esteem, mood, and quality of life** (Q13 to Q16)

Each factor demonstrated acceptable to excellent internal consistency, with Cronbach’s alpha coefficients (α) ranging from 0.68 to 0.92.

**2.3.3 Scoring procedure**

For each participant, the following scores were computed:

• **Subscale scores** (F1 to F4): Calculated as the mean of the items within each respective dimension.
• **A global score:** Computed as the mean of all 16 items.

To facilitate comparison across dimensions, all scores were transformed to a 0–100 scale using the following formula:

$$\text{transformed score}=\frac{\mathrm{raw}\;\text{mean score}}{5}\times 100$$

Higher transformed scores reflect greater negative body image impact.

Data processing was conducted using IBM SPSS Statistics (Version 26.0; IBM Corp, Armonk, NY, USA) and standardized syntax was applied to ensure consistency in scoring across the entire sample.

### 2.4 Data collection

Data collection was conducted during hospital consultations for each included woman followed for gynecological and breast cancers.

French and Arabic versions of the questionnaire were specifically developed for the needs of this study.

Data were collected through self-administered questionnaires in a private setting, with assistance available if needed. The primary goal of the questionnaire was to assess body image. Demographic (marital status, education level, socioeconomic level, ethnicity …) and clinical data (primary cancer diagnosis, time since diagnosis, current treatment modality [chemotherapy/radiation/surgery/hormonal therapy], treatment history …) were also recorded.

To preserve participant anonymity, no personally identifiable information, such as names or contact details, was collected.

### 2.5 Statistical methods

Descriptive statistics were used to summarize participant characteristics and score distributions. Categorical variables (e.g., cancer type, marital status) are summarized using frequencies and percentages. Continuous variables (age, scale scores) are reported as means ± standard deviations (SD) if normally distributed, or medians and interquartile ranges (IQR) if non-normal.

Internal consistency of the newly developed scale (total score and identified subscales) was assessed using Cronbach’s alpha. An alpha coefficient ≥ 0.70 was deemed acceptable for research purposes. ^18^

Convergent validity was evaluated using Spearman’s rank-order correlations (ρ) between the new scale’s total score and validated measures: the FSFI ^15^ and BESAA. ^16^ Moderate-to-strong positive correlations (ρ ≥ 0.40) were hypothesized a priori based on conceptual overlap.

All analyses were performed using IBM SPSS Statistics (Version 26.0; IBM Corp, Armonk, NY, USA) for data management, descriptive statistics, correlation analysis, and non-parametric tests.

Exploratory Factor Analysis (EFA) and Cronbach’s alpha computation were conducted in JASP (Version 0.18.1; JASP Team, Amsterdam, Netherlands).

Prior to analysis, data were screened for missing values, outliers, and normality. Missing item-level data on the new scale were handled using pairwise deletion during EFA and reliability analysis. Total/subscale scores were computed only for participants with ≤20% missing items on the respective scale; otherwise, scores were treated as missing. Continuous variables were assessed for normality using Shapiro-Wilk tests (p < .05 threshold) and visual inspection of Q-Q plots.

The analysis followed several steps ^19, 20^:

**2.5.1 Assessment of suitability for exploratory factor analysis:**

The adequacy of the data for factor analysis was evaluated using the Kaiser-Meyer-Olkin ^21^ (KMO) measure and Bartlett’s Test of Sphericity. A KMO value above 0.80 was considered very good, while a significant Bartlett’s test (p < .05) indicated that correlations between items were sufficient to proceed with factor extraction.

**2.5.2 Exploratory factor analysis:**

EFA was performed on the new scale’s items using Principal Axis Factoring (PAF) to extract common variance. Factor retention was determined by:

• **Suitability:** KMO measure >0.80 and Bartlett’s Test of Sphericity (p < .05).
• **Extraction Criteria:** Eigenvalues >1.0, scree plot inflection point, and factor interpretability.
• **Rotation:** Promax rotation (κ = 4) allowing correlated factors.

**2.5.3 Factor retention criteria:**

Items with pattern matrix loadings ≥0.40 on a primary factor and cross-loadings <0.30 were retained. Items with uniqueness >0.90 or communality <0.20 were reviewed for conceptual misfit.

**2.5.4 Measure of Sampling Adequacy (MSA):**

The MSA was calculated for each item to assess its individual contribution to the overall factor model. Values above 0.50 were deemed acceptable. ^22^

**2.5.5 Interpretation of factors:**

Factors were labeled based on items loading ≥0.50.

Differences in scale scores across categorical clinical variables (e.g., cancer type [Grouped as: Breast vs. Gynecological vs. Colorectal], active treatment [Yes/No]) were tested using:

• Mann-Whitney U test (2 groups)
• Kruskal-Wallis H test (>2 groups) with Dunn-Bonferroni post-hoc tests and effect size η ^2^_H_
• Significance was set at p < 0.05 (two-tailed). Effect sizes were interpreted as: r/η ^2^_H_ ≥ 0.10 (small), ≥0.30 (medium), ≥0.50 (large).

### 2.6 Ethical considerations

The study protocol was approved on 13 February 2025 by the institutional ethics committee of Charles Nicolle Hospital, Tunis, Tunisia before conducting the study with approval number FWA 00032748- IORG0011243.

Verbal informed consent was selected instead of written consent due to the specific context of the study population.

Data were collected from population considered vulnerable due to both their cancer diagnosis and the psychological impact of treatment. Requesting written consent in this setting risked creating additional stress or discouraging participation, particularly among women with lower literacy levels.

This approach was reviewed and approved by the Institutional Ethics Committee of Charles Nicolle Hospital as the study involved minimal risk, collected no identifying or sensitive information, and ensured complete anonymity of responses.

All participants received detailed information regarding the study’s objectives. They were clearly informed of their right to withdraw from the study at any point without having to justify their decision. Confidentiality of all participant data was strictly preserved throughout the study.

## 3. Results

This study analyzed data from 101 female cancer patients, examining sociodemographic and clinical characteristics.

Patients ranged in age from 21 to 68 years (mean 44.9, median 44). The cohort consisted primarily of married women (86.1%) with secondary education (40.6%). Most patients belonged to medium socioeconomic status (74.3%) and resided in urban areas (57.4%). Language preferences were Arabic (64.4%) and French (35.6%) ( Table 1).

**Table 1.** Sociodemographic characteristics of the study population.

Variable	Category	Frequency (n = 101)	Percentage (%)
**Language Preference**	**Arabic**	65	64.4
	**French**	36	35.6
**Marital Status**	**Married**	87	86.1
	**Single**	8	7.9
	**Divorced**	4	4.0
	**Widowed**	2	2.0
**Education Level**	**Illiterate**	5	5.0
	**Primary**	25	24.8
	**Secondary**	41	40.6
	**University**	30	29.7
**Socioeconomic Level**	**Low**	17	16.8
	**Medium**	75	74.3
**Residence**	**High**	9	8.9
	**Urban**	58	57.4
	**Rural**	43	42.6

Breast cancer accounted for 45.5% of cases, followed by endometrial (25.7%) and cervical cancers (13.9%). Family history of cancer was present in 48.5% of patients. Treatment modalities included combination therapies (67.3%), primarily surgery with adjuvant chemotherapy or radiation, while 14.9% received surgery alone. The most frequent treatment facilities were public hospitals: Salah Azaiez (15.8%) and Jendouba (16.8%), with 8.9% treated at private clinics ( Table 2).

**Table 2.** Personal and family history of cancer among the study population.

Variable	Category	Frequency (n = 101)	Percentage (%)
**Cancer Type**	**Breast Cancer**	46	45.5
	**Endometrial Cancer**	26	25.7
	**Cervical Cancer**	14	13.9
	**Ovarian Cancer**	11	10.9
	**Other Combinations**	4	4
**Family History of Cancer**	**Yes**	49	48
	**No**	52	51.5
**Treatment Received**	**Surgery Only**	15	14.9
	**Adjuvant Chemotherapy**	18	17.8
	**Various Combinations**	68	67.3
**Hospital of Treatment**	**Salah Azaiez**	16	15.8
	**Jendouba Hospital**	17	16.8
	**Private Clinics**	9	8.9
	**Other Hospitals**	59	58.4

Reproductive history showed a mean of 2.7 pregnancies and 2.1 children per patient. The mean time since treatment completion was 20.1 months (median 12 months, range 3-72 months) ( Table 3).

**Table 3.** Quantitative characteristics of the study population.

Variable	Mean	Median	Range	Standard deviation
**Age (years)**	44.92	44	21-68	11.15
**Number of pregnancies**	2.66	2	0-16	2.09
**Number of children**	2.06	2	0-5	1.24
**Time since treatment ended (months)**	20.09	12	3-72	16.01

These findings characterize a predominantly middle-aged, married patient population with medium socioeconomic status, most frequently diagnosed with breast cancer and treated at public hospitals with multimodal approaches. The substantial proportion with family cancer history (48.5%) suggests potential genetic factors requiring further investigation. The follow-up duration range (3-72 months) permits analysis of both short- and long-term outcomes.

### 3.1 Exploratory factor analysis

An EFA was conducted to examine the underlying structure of the composite score following oncological treatment. All comprehensive details of the EFA/CFA conducted in this study can be found at the following link: https://doi.org/10.7910/DVN/ZGECTK. ^17^

The data showed strong suitability for factor analysis (KMO = 0.854; Bartlett’s test χ ^2^ = 872.986, df = 153, *p* < .001).

Two interpretable factors were extracted using Promax rotation, accounting for 43.8% of the total variance:

• **Factor 1**, labeled *Sexual and Relational Dimension*, included items related to sexual desire, scar-related discomfort, relationship satisfaction, and perceived desirability.
• **Factor 2**, labeled *Emotional and Self-Esteem Dimension*, grouped items referring to anxiety, attractiveness, physical weakness, and self-confidence.

Items Q6 “Since your diagnosis, has your perception of your body evolved?” and Q18 “Have you discussed your concerns about body image with your care team?” showed weak loadings and high uniqueness, suggesting they are poorly represented by the extracted factor structure.

All other items had acceptable Measures of Sampling Adequacy (MSA > 0.5), with the highest for items Q5 “Do you feel that your body is weaker or less capable than before?”, Q9 “Does your body image affect your participation in social activities”, and Q15 “Do you feel anxious or depressed because of the physical changes related to your illness or treatment?”.

Following the removal of problematic items 17 “Have you received any psychological or aesthetic support to help you accept the changes in your physical appearance?” and 18, we conducted a CFA to test the hypothesized 4-factor structure comprising 16 items. The model demonstrated acceptable to good fit across multiple indices. While the significant χ ^2^ statistic (χ ^2^ (98) = 165.087, p < .001) suggested some model-data discrepancy, this is expected with larger samples. More importantly, the CFI (0.914) and SRMR (0.069) met excellent fit thresholds, while the TLI (0.895) and RMSEA (0.082 [0.060-0.104]) approached or met acceptable levels. The GFI (0.830) indicated moderate fit, representing a notable improvement over previous specification.

Reliability analysis revealed strong internal consistency for three factors:

• Sexual Desirability showed excellent reliability (ω = 0.918, AVE = 0.741),
• Followed by Self-Confidence/QoL (ω = 0.786, AVE = 0.549),
• And Social exposure (ω = 0.737, AVE = 0.470).

While Appearance Concerns showed marginally acceptable reliability (ω = 0.704, AVE = 0.377), we retained this factor due to its theoretical importance in the measurement model.

One persistent concern emerged regarding item 6, which demonstrated poor factor explanation (R ^2^ = 0.033) and non-significant loading (β = -0.188, p = 0.089). This item had shown consistent problems in prior analyses, suggesting it may not adequately capture the intended construct.

These results support the validity of the refined 4-factor structure while suggesting potential for further improvement through removal of item 6. Such modification would likely enhance the model’s psychometric properties by improving factor coherence and increasing average variance extracted, while maintaining comprehensive coverage of the theoretical domains. The current solution nevertheless represents a statistically and conceptually sound measurement model for assessing body image concerns in this population.

The analysis revealed strong and statistically significant correlations (all p < .001) between all four body image factors and the global score. Appearance concerns (F1) showed particularly strong associations with Self-esteem (F4; ρ = .639) and Social exposure (F2; ρ = .607), suggesting these dimensions share considerable variance in how individuals experience body image. The Sexual desirability factor (F3) demonstrated slightly lower but still substantial correlations with other factors (range: ρ = .465-.597), indicating it captures both related and unique aspects of body image.

The global 4D-BISC showed the strongest relationships with all individual factors (ρ = .784-.830), with the highest correlation occurring with Social exposure (F2; ρ = .830).

### 3.2 External validation

The correlation analysis revealed significant associations between participants’ body image perceptions and their sexual functioning across multiple domains ( Table 4).

**Table 4.** Spearman's correlations between body image factors and sexual function outcomes.

Sexual function domain	F1: Appearance concerns	F2: Social exposure	F3: Sexual desirability	F4: Self-esteem	Global score
**Sexual desire**	.547 ***	.605 ***	.706 ***	.542 ***	.747 ***
**Arousal**	.492 ***	.581 ***	.569 ***	.545 ***	.669 ***
**Lubrication**	.488 ***	.565 ***	.533 ***	.536 ***	.647 ***
**Orgasm**	.450 ***	.518 ***	.488 ***	.521 ***	.601 ***
**Satisfaction**	.420 ***	.455 ***	.465 ***	.488 ***	.558 ***
**Sexual pain**	.494 ***	.578 ***	.545 ***	.528 ***	.655 ***
**FSFI Total**	.521 ***	.595 ***	.583 ***	.567 ***	.693 ***
**BESAA Score**	-.470 ***	-.426 ***	-.386 ***	-.458 ***	-.528 ***

All values represent Spearman's rho coefficients.

(F: Factor)

* p < .05,

** p < .01,

*** p < .001.

All four body image factors - Appearance Concerns (F1), Social Exposure (F2), Sexual Desirability (F3), and Self-Esteem (F4) - demonstrated moderate to strong positive correlations with various aspects of sexual function (all p < .001). These consistent patterns suggest that greater body image concerns tend to co-occur with more reported sexual difficulties.

The strongest positive relationships emerged for sexual desire, which showed particularly robust associations with both the Sexual Desirability factor (ρ = .706) and the global 4D-BISC (ρ = .747). This indicates that women’s perceptions of their sexual attractiveness may be especially relevant for maintaining sexual interest.

Similarly, the global 4D-BISC showed strong correlations with sexual pain (ρ = .655) and overall sexual function as measured by the FSFI total score (ρ = .693), suggesting that body image disturbances may contribute to both physical and psychological aspects of sexual dysfunction.

Notably, the BESAA showed an inverse pattern of relationships. Lower body esteem scores (reflecting more negative body appraisal) were significantly associated with greater body image concerns across all factors (ρ = -.386 to -.470) and poorer sexual function in most domains (ρ = -.201 to -.295). The exception was sexual satisfaction, which did not show a significant correlation with body esteem (ρ = -.083, p = .409), possibly indicating that satisfaction depends on additional factors beyond body image alone.

### 3.3 Comparative study

Table 5 presents the comparative analysis among the study population.

**Table 5.** Comparative analysis among the study population.

Characteristic	Category	F1		F2		F3		F4		Global score	
		Median	p-value	Median	p-value	Median	p-value	Median	p-value	Median	p-value
**Education Level**	**Illiterate**	50	0.08	60	0.149	60	0.866	40	0.136	53.8	0.168
	**Primary**	33.3		26.7		25		40		33.8	
	**Secondary**	40		33.3		45		40		40	
	**University**	33.3		30		37.5		36.7		33.8	
**Marital Status**	**Single**	40	0.311	30	0.275	0	0.018*	36.7	0.814	26.3	0.418
	**Divorced**	25		20		25		26.7		26.3	
	**Married**	36.7		40		45		40		38.8	
	**Widowed**	25		30		0		23.3		19.4	
**Socioeconomic Status**	**Low**	40	0.104	40	0.078	45	0.514	40	0.024*	38.8	0.073
	**High**	33.3		33.3		35		40		33.8	
	**Middle**	36.7		26.7		40		40		37.5	
**Cancer Type**	**Endometrial**	35	0.244	40	0.018*	47.5	0.235	46.7	0.013*	42.5	0.039*
	**Ovarian**	40	0.646	40	0.273	60	0.001*	46.7	0.301	45	0.049*
	**Cervical**	36.7	0.948	40	0.305	40	0.327	40	0.512	38.8	0.408
	**Breast**	36.7	0.360	26.7	0.006*	30	0.002*	33.3	0.004*	30	0.003*
**Residence**	**Rural**	36.7	0.351	40	0.497	35	0.077	40	0.592	35	0.336
	**Urban**	36.7		33.3		45		40		38.8	
**Family Cancer History**	**No**	36.7	0.209	26.7	0.028*	35	0.009*	33.3	0.009*	33.8	0.008*
	**Yes**	36.7		40		50		46.7		40	

**F1**: Appearance Concerns, **F2:** Social Exposure, **F3**: Sexual Desirability, **F4:** Self-Esteem

(F: Factor).

Illiterate patients reported the highest levels of appearance-related concerns (median = 50) and social exposure anxiety (median = 60), though these education-related differences only approached statistical significance (p = 0.08 for appearance). Those with primary education showed the most negative body image profiles overall, particularly regarding sexual desirability (median = 25).

Socioeconomic status significantly impacted self-esteem (p = 0.024), with low-income patients maintaining better self-image (median = 40) compared to middle and high-income groups.

Marital status emerged as an important factor, particularly for sexuality-related body image (p = 0.018). Widowed and single patients reported the most severe concerns about sexual desirability (median = 0), while married participants showed the most positive body image profiles overall (global median = 38.8). These results suggest that relationship status may serve as either a protective or risk factor for body image disturbances.

The most pronounced differences appeared when examining cancer types. Breast cancer patients demonstrated significantly poorer body image across all domains (global p = 0.003), with particularly low scores for social exposure (median = 26.7, p = 0.006) and sexual desirability (median = 30, p = 0.002). In contrast, ovarian cancer patients reported the highest levels of sexual concerns (median = 60, p = 0.001), while endometrial cancer patients showed relatively better self-esteem (median = 46.7, p = 0.013). These cancer-specific patterns highlight the need for tailored interventions addressing the unique body image challenges associated with different cancer diagnoses.

Patients with a family history of cancer showed significantly better body image across nearly all factors (global p = 0.008), particularly regarding sexual desirability (median = 50 vs 35 for those without family history, p = 0.009). This finding suggests that prior experience with cancer in the family may foster more adaptive body image perceptions. Urban-rural differences approached significance for sexual concerns (p = 0.077), with urban residents reporting higher scores, possibly reflecting differing cultural norms around body image.

The correlations between body image factors and patient characteristics are summarized in Table 6.

**Table 6.** Correlations between body image factors and patient characteristics.

Body image dimension	Age	Number of pregnancies	Number of children	Time since treatment completion
**F1: Appearance Concerns**	-0.184 (p = 0.066)	0.010 (p = 0.917)	-0.028 (p = 0.783)	0.083 (p = 0.408)
**F2: Social Exposure**	-0.075 (p = 0.454)	-0.007 (p = 0.947)	0.056 (p = 0.579)	0.151 (p = 0.131)
**F3: Sexual Desirability**	-0.098 (p = 0.331)	0.088 (p = 0.381)	0.004 (p = 0.968)	**0.207 * (p = 0.038) **
**F4: Self-Esteem **	-0.101 (p = 0.315)	0.015 (p = 0.879)	0.036 (p = 0.720)	0.046 (p = 0.649)
**Global Score**	-0.144 (p = 0.152)	0.040 (p = 0.689)	0.015 (p = 0.880)	0.159 (p = 0.111)

All values represent Spearman's rho coefficients.

* p < .05,

** p < .01,

*** p < .001.

Regarding age, all correlations with body image dimensions were weak and negative, but none reached statistical significance. This indicates that older patients did not significantly differ in their perception of appearance, exposure, sexuality, self-esteem, or global body image compared to younger patients.

For the number of pregnancies, correlations were negligible and non-significant across all dimensions, suggesting that parity did not influence body image perception in this sample.

Similarly, the number of children showed no meaningful correlations with body image scores, indicating no significant relationship between parental status and body image.

In contrast, time since treatment completion showed a significant positive correlation with the sexuality dimension (r = 0.207, p = 0.038), suggesting that sexual well-being improved with longer time elapsed after treatment. No significant associations were found with appearance, exposure, self-esteem, or the global body image score.

## 4. Discussion

### 4.1 Comparative analysis of body image assessment in breast cancer patients

Our findings demonstrate both convergence and divergence with existing literature on body image assessment in breast cancer populations.

Like Baxter et al. and Gonçalves et al., found that body image is a multidimensional construct requiring comprehensive assessment. ^23, 24^ However, while their Body Image after Breast Cancer Questionnaire (BIBCQ) identified six factors, our analysis revealed four primary dimensions (Appearance concerns, Social exposure, Sexual desirability, and Self-Esteem), suggesting potential cultural or methodological differences in factor structure.

The reliability coefficients in our study (ω = 0.704-0.918) compare favorably with those reported in multiple validations of the BIBCQ (α = 0.77-0.87) and the Body Image, Sexuality, and Breast Cancer questionnaire (BAS-BC) (α = 0.91), indicating similarly robust measurement properties. Notably, our Sexual Desirability factor showed particularly strong reliability (ω = 0.918), paralleling findings from Zhou et al. ^25^ where sexuality-related subscales demonstrated high internal consistency (α up to 0.88).

Our results showing significant body image differences by cancer type (particularly poorer outcomes for breast cancer patients) align with Guedes et al.’s report of 74.8% body image dissatisfaction post-treatment. ^26^ However, we extended these findings by identifying specific vulnerabilities in sexual body image (median = 30) and exposure concerns (median = 26.7) among breast cancer patients, providing more nuanced clinical targets.

The significant improvement in sexuality-related body image over time post-treatment (r = 0.207, p = 0.038) in our study complements Sema Koçan et al.’s systematic review findings that body image satisfaction improves over time after mastectomy. ^27^ However, our identification of age-related trends (older patients reporting fewer appearance concerns) adds new dimensions to their observation that younger women face greater body image challenges.

Unlike Derbis and Czerwik’s Polish adaptation that found limited BIBCQ correlations with sexual functioning, we observed robust associations between our Sexual Desirability factor and time since treatment. ^28^ This discrepancy may reflect cultural differences in sexuality expression or our focus on temporal rather than functional aspects.

### 4.2 Study strengths and limitations

While our study shares the commonly cited limitation of limited sample size, as highlighted by Zhou et al., ^25^ it advances existing research in several meaningful ways:

- It identifies time-sensitive patterns in body image recovery, offering insights into the temporal dynamics of patient adjustment,
- It highlights differential impacts on body image and sexuality across various cancer types, rather than focusing exclusively on breast cancer,
- It introduces validated and concise assessment dimensions, supporting more efficient and targeted clinical evaluation.

Moreover, our findings address key gaps outlined in the review by Muzzatti and Annunziata. ^29^ Whereas they observed that most existing tools are narrowly tailored to breast cancer populations in English-speaking countries, our study demonstrates broader cultural and linguistic applicability across different cancer types in a non-English-speaking context.

Additionally, the four-factor structure proposed in our analysis offers a more parsimonious and clinically practical alternative to the eight different instruments reviewed in their study, thus contributing to simplified and streamlined assessments in routine oncology care.

### 4.3 Clinical implications

Our findings underscore the importance of targeted interventions to address sexual body image concerns, particularly among breast cancer patients in the early post-treatment phase, when vulnerability appears to be highest.

The validated dimensions identified in this study offer a theoretical and practical framework for culturally adapted assessments, helping to overcome limitations previously noted in cross-cultural validation studies.

The observed temporal patterns in body image adjustment provide critical insights into the optimal timing for intervention delivery, supporting the development of time-sensitive psychosocial support programs.

### 4.4 Recommendations for future research

Future research should seek to integrate our dimensional approach with existing comprehensive psychosocial evaluation frameworks, as proposed in prior studies, to facilitate the development of more personalized and context-sensitive intervention strategies.

Given the robust psychometric properties of our 4D-BISC, further studies are warranted to pursue its cross-cultural adaptation and validation. This would help address the ongoing need for diverse, culturally appropriate assessment tools in psycho-oncology, as consistently emphasized in the literature.

## 5. Conclusions

Our findings indicate that the 4D-BISC is a reliable and valid instrument for assessing body image and sexual well-being in women with breast and gynecological cancers. These aspects play a crucial role in patients’ psychological recovery and overall quality of life, especially during the post-treatment period. As such, the 4D-BISC represents a valuable tool for investigating the factors influencing body image in cancer patients, with important implications for psychosocial care and supportive interventions in oncology.

The 4D-BISC has the potential to serve as a cross-culturally adaptable, psychometrically robust tool for both research and clinical practice.

## Ethical considerations

We affirm that we have reviewed the Journal’s ethical publication guidelines and confirm that this report adheres to those standards.

The study protocol received approval from the Institutional Ethics Committee of Charles Nicolle Hospital, Tunis, Tunisia, on 13 February 2025, prior to the initiation of the study (approval number: FWA 00032748 – IORG0011243).

## Consent to participate

Given the minimal-risk, observational design of the study and the inclusion of a population considered vulnerable due to both their cancer diagnosis and the psychological impact of treatment, informed consent was obtained orally rather than in writing.

This approach allowed explanations to be provided in the participant’s preferred language (Arabic or French), ensured full comprehension despite variable literacy levels, and avoided cultural barriers associated with signing formal documents.

Each participant’s decision was carefully documented by the investigators, and the procedure was reviewed and explicitly approved by the Institutional Ethics Committee of Charles Nicolle Hospital, Tunis, Tunisia

All participants were informed about the objectives of the study and invited to participate upon providing their consent. They were made aware of their right to withdraw from the study at any time without the need to provide a reason. Participant confidentiality was maintained throughout the duration of the study.

## Data Availability

All data sets can be assessed and all study findings reported in the article are shared via Harvard Dataverse: “Beyond Scars: Development and Validation of a Four-Dimensional Body Image Score for Women with Breast and Gynecological Cancers - A Tunisian Multicenter Study”, https://doi.org/10.7910/DVN/ZGECTK. ^17^ This project contains the following:
- Dataset Body image - Study Findings Body image (English) - CFA without Q6, Q17, Q18 - CFA without Q17 - EFA - CFA (Reliability analysis 1) - CFA (Reliability analysis 2) Harvard Dataverse: “Beyond Scars: Development and Validation of a Four-Dimensional Body Image Score for Women with Breast and Gynecological Cancers - A Tunisian Multicenter Study”, https://doi.org/10.7910/DVN/ZGECTK. ^17^ This project contains the following:
- Initial Questionnaire (in English) - Initial Questionnaire (in French) - Initial Questionnaire (in Arabic) - The 4D-BISC Final (in English) - The 4D-BISC Final (in French) - The 4D-BISC Final (in Arabic) Data are available under the terms of the Creative Commons Zero “No rights reserved” data waiver (CC0 1.0 Public domain dedication).
