# Supplementary material for: Beyond Scars: Development and Validation of a Four-Dimensional Body Image Score for Women with Breast and Gynecological Cancers - A Tunisian Multicenter Study
Source: F1000Res. 2025 Sep 3;14:863. [Version 1] doi: 10.12688/f1000research.169080.1 (PMC13044537; doi:10.12688/f1000research.169080.1)
Supplement: Peer review history

# Reviewer report: Reviewer response for version 1

**DOI:** 10.5256/f1000research.186365.r470864

**Published:** 2026-04-01

**Authors:** Massimo Tusconi

**Affiliations:** 1 University of Cagliari, Cagliari, Italy

I appreciate the opportunity to review the manuscript entitled: "Beyond Scars: Development and Validation of a Four-Dimensional Body Image Score for Women with Breast and Gynecological Cancers - A Tunisian Multicenter Study".

 The manuscript addresses a clinically relevant and timely topic in psycho-oncology, focusing on the multidimensional assessment of body image in women with cancer. I commend the authors for describing this critical and timely issue. The paper is interesting and well-written; however, I would like to highlight some issues that merit revision:

 - The manuscript presents an inconsistency between the exploratory factor analysis (two-factor solution) and the confirmatory factor analysis (four-factor model). I kindly ask the authors to clarify the theoretical and statistical rationale for retaining the four-factor structure despite the EFA findings.

 - The sample size (n = 101) appears limited for performing both EFA and CFA on the same dataset. Please consider discussing the potential risk of overfitting and, if possible, justify this approach or suggest future validation strategies.

 - Item 6 shows poor factor loading and low explanatory power, yet it is retained in the final model. I ask the authors to provide a stronger justification for its inclusion or to consider its removal and report alternative model fit indices.

 - The use of verbal informed consent is appropriately justified; however, I kindly ask the authors to explicitly acknowledge potential limitations regarding traceability and documentation in the limitations section.

 - The absence of potential confounders (e.g., comorbidities such as diabetes or HIV, cancer stage, treatment type) may affect the interpretation of results. Please consider addressing these variables or discussing their impact.

 - While the correlations with FSFI and BESAA are strong and represent a key strength of the study, I ask the authors to elaborate further on the clinical implications of these findings, particularly in terms of intervention development and patient management.

 Overall, the manuscript is interesting because it proposes a novel and potentially useful tool for assessing body image in cancer patients, contributing to a more integrated psychosocial evaluation. I look forward to the authors’ responses to these queries in order to improve the manuscript’s clarity and impact.

Is the work clearly and accurately presented and does it cite the current literature?

Yes

If applicable, is the statistical analysis and its interpretation appropriate?

Yes

Are all the source data underlying the results available to ensure full reproducibility?

Yes

Is the study design appropriate and is the work technically sound?

Yes

Are the conclusions drawn adequately supported by the results?

Yes

Are sufficient details of methods and analysis provided to allow replication by others?

Yes

Reviewer Expertise:

Mood, Bipolar, Psychosis, Biomarkers

I confirm that I have read this submission and believe that I have an appropriate level of expertise to confirm that it is of an acceptable scientific standard, however I have significant reservations, as outlined above.

# Reviewer report: Reviewer response for version 1

**DOI:** 10.5256/f1000research.186365.r470867

**Published:** 2026-03-28

**Authors:** Amayra Tannoubi

**Affiliations:** 1 High Institute of Sport and Physical Education of Gafsa, University of Gafsa, Gafsa, Tunisia

Abstract

 The abstract is structured with clear Background, Methods, Results, and Conclusion sub-sections and accurately reflects the content of the manuscript. The key findings are well-summarized. However, the abstract mentions a "robust 4-factor structure" confirmed by CFA while the Results section reveals that the TLI (0.895) did not meet the commonly accepted threshold of ≥ 0.95 and the RMSEA (0.082) only approached acceptable levels — the abstract therefore overstates the fit quality and should be revised to more accurately qualify these values.​

 Introduction

 The Introduction is well-written, clinically motivated, and builds a convincing case for the need for an integrative, cancer-specific body image tool. The critique of existing instruments (BESAQ, EORTC QLQ) is specific and relevant. However, the final two paragraphs are partially repetitive, restating the same rationale and objective with slightly different wording; these should be consolidated into one focused paragraph ending with a single, clearly stated research objective.​

 Methods

**Study design and setting:** 

 The multicenter design across six facilities of varying levels (regional and tertiary) is a strength, improving ecological validity. The two-month data collection window (March–April 2025) is narrow and should be briefly discussed in terms of potential seasonal or contextual biases.​

**Study population:** 

 Inclusion and exclusion criteria are clear. However, no potential confounders are controlled or even mentioned — notably, HIV status and diabetes, which are documented risk factors for gynecological cancers and may independently affect body image, sexual function, and self-esteem, are not addressed.​

**Scale development:** 

 The description of item derivation from FSFI and BESAA is adequate, but the content validity process (e.g., expert panel review, cognitive debriefing, pilot testing) is only cursorily described. Given that the scale is a novel instrument, a more explicit account of item generation and refinement procedures is expected.​

**Consent procedure:** 

 The use of verbal rather than written informed consent is justified on grounds of population vulnerability and low literacy — and this justification was reviewed and approved by the ethics committee. However, this introduces a potential limitation in consent documentation and traceability that should be acknowledged in the Limitations section.​

**Statistical methods:** 

 The analytic pipeline (EFA → CFA → reliability → convergent validity → comparative analysis) is well-described and appropriate. The use of Promax rotation, PAF extraction, and Rasch-complementary approaches is methodologically sound. However, the analysis uses both EFA and CFA on the same dataset of only 101 participants — this is a well-known limitation (overfitting risk) that is insufficiently addressed. A split-sample or cross-validation strategy would have been preferable, or at minimum, this should be prominently discussed.​

 Results

**Descriptive statistics:** 

 Participant characteristics are well-described across three tables. The sample is predominantly married (86.1%), medium socioeconomic status (74.3%), and breast cancer (45.5%), which limits generalizability to more diverse populations.​

**EFA:** 

 The KMO (0.854) and Bartlett's test (p < .001) confirm data suitability. However, the EFA extracted only two factors accounting for 43.8% of variance, while the subsequent CFA tests a four-factor structure — this inconsistency between EFA and CFA factor solutions is a fundamental tension in the manuscript that is not adequately explained or resolved.​

**CFA:** 

 The fit indices are mixed: CFI (0.914) and SRMR (0.069) are acceptable, but TLI (0.895) falls below the conventional 0.95 threshold and RMSEA (0.082) approaches but does not comfortably meet the ≤ 0.08 criterion. The GFI (0.830) indicates only moderate fit. These results should be described as "acceptable" rather than "robust," and alternative model specifications (e.g., with item 6 removed) should be formally tested and reported.​

**Item 6:** This item shows non-significant loading (β = −0.188, p = 0.089) and very low R² (0.033). The authors flag it as a persistent concern but retain it in the final model—no clear rationale for retention is provided beyond noting its theoretical importance. If it is to be retained, convergent validity evidence for this specific item is needed.​

**External validation:** 

 The Spearman correlations with FSFI and BESAA are strong, consistent, and well-presented in Table 4. The inverse relationship with BESAA scores is correctly interpreted. This is the strongest section of the results.​

**Comparative analysis:**

  The group comparisons across cancer type, marital status, and socioeconomic status are informative and clinically relevant. Effect sizes (η²H) are reported for some but not all comparisons — they should be consistently provided for all significant results.​

 Discussion

 The discussion is organized into four clear subsections and engages substantively with the existing literature. The comparison with BIBCQ, BAS-BC, and Zhou et al.'s findings is appropriate and well-contextualized. However, the fundamental inconsistency between the two-factor EFA solution and the four-factor CFA model is not directly addressed—this discrepancy is the most critical methodological issue in the paper, and the Discussion should confront it explicitly. The interpretation of the family cancer history finding (better body image in patients with a family history) is intriguing but underdeveloped; a more careful interpretation of this counterintuitive result is needed.​

 Conclusion

 The conclusion is appropriately brief and aligned with the findings. The claim that the 4D-BISC has "potential to serve as a cross-culturally adaptable, psychometrically robust tool" is premature given the small sample, the single-country design, and the mixed CFA fit—this claim should be toned down to reflect the preliminary nature of the validation.​

 Limitations

 A dedicated Strengths and Limitations subsection (4.2) is included, which is commendable. However, the most critical limitations are underplayed or absent:​

- The EFA–CFA discrepancy (2 vs. 4 factors) on the same small sample is not mentioned
- Verbal-only consent and its implications for traceability are not discussed
- Absence of test-retest reliability data is not acknowledged
- No discrimination between cancer stage, treatment duration, or specific surgical procedures (e.g., mastectomy vs. lumpectomy), all of which could confound body image scores

Is the work clearly and accurately presented and does it cite the current literature?

Yes

If applicable, is the statistical analysis and its interpretation appropriate?

Yes

Are all the source data underlying the results available to ensure full reproducibility?

Partly

Is the study design appropriate and is the work technically sound?

Partly

Are the conclusions drawn adequately supported by the results?

Partly

Are sufficient details of methods and analysis provided to allow replication by others?

Partly

Reviewer Expertise:

Validation

I confirm that I have read this submission and believe that I have an appropriate level of expertise to confirm that it is of an acceptable scientific standard, however I have significant reservations, as outlined above.

# Reviewer report: Reviewer response for version 1

**DOI:** 10.5256/f1000research.186365.r442292

**Published:** 2026-01-05

**Authors:** Mathew Olumide Adebisi

**Affiliations:** 1 Afe Babalola University, Aye, Nigeria

General comments: The study delved into important part of psycho-oncology in breast and gynaecological malignancies.. Some minor corrections/clarifications/observations are noted.

 Methods: 1. Some medical conditions are noted to be risk factors for gynaecological cancers especially cervical cancers. Did the authors exclude those with diabetes mellitus and HIV from the study? These two conditions among others can impact on the novel/proposed 4D-Body Image Scoring system as confounders.

              2. Authors did not mention the use of interpreters in the methodology, yet they included non-English-speaking population. I suggest authors add the role of the interpreters when needed to the methodology. 

 Results: Table 1- the level of education can be classified as illiterate, primary, secondary and tertiary.  Authors can also substitute  tertiary with university or equivalents. Socioeconomic status not well aligned in the table. 

 Consent to participate: Authors mentioned that the objectives were discussed with the participants. This can lead to biases in the responses. Truly, participants or respondents need to have adequate information about the study but detailing the objectives to them can influence the responses/answers to the questions. Objectives are the parts of the important meats of any study. These aspect should be reconsidered.

 Final note: If the above suggestions/corrections are accepted by the authors, revisions can be carried out to reflect the acceptance.

Is the work clearly and accurately presented and does it cite the current literature?

Partly

If applicable, is the statistical analysis and its interpretation appropriate?

Yes

Are all the source data underlying the results available to ensure full reproducibility?

Partly

Is the study design appropriate and is the work technically sound?

Yes

Are the conclusions drawn adequately supported by the results?

Yes

Are sufficient details of methods and analysis provided to allow replication by others?

Yes

Reviewer Expertise:

Obstetrics and Gynaecology

I confirm that I have read this submission and believe that I have an appropriate level of expertise to confirm that it is of an acceptable scientific standard.

# Response

**Published:** 2026-01-06

**Authors:** Narjes karmous

**Affiliations:** University of Tunis El Manar Faculty of Medicine of Tunis, Tunis, Tunis, Tunisia

Dear Dr ADEBISI,

 We sincerely thank you for your careful review of our manuscript and for your positive overall evaluation and approval of the work.

 We are particularly grateful for your constructive comments and thoughtful suggestions regarding the methodology, presentation of results, and ethical considerations. We fully agree that the proposed clarifications - including those related to potential confounders, the role of interpreters, refinement of sociodemographic variables, and the consent process- will further enhance the clarity, methodological rigor, and reproducibility of the manuscript.

 We would like to confirm that all the points you raised will be carefully addressed and incorporated in the revised version. At this stage, we are awaiting the feedback from the second reviewer, after which we will proceed with a consolidated revision to ensure coherence and completeness across all sections of the manuscript.

 Once again, we sincerely appreciate your time, expertise, and valuable contribution to improving the quality of our work.

 Kind regards,

 Narjes
